# Problem gambling among ethnic minorities: results from an epidemiological study

**DOI:** 10.1186/s40405-017-0027-2

**Published:** 2017-09-07

**Authors:** Kyle R. Caler, Jose Ricardo Vargas Garcia, Lia Nower

**Affiliations:** Center for Gambling Studies, School of Social Work, Rutgers University, 536 George Street, New Brunswick, NJ 08901 USA

## Abstract

A few studies have examined gambling behavior and problem gambling among minorities and reported higher rates of both participation and gambling problems among particular minority groups in comparison to Whites who gamble. The present study utilized a representative, epidemiological sample of adults in New Jersey to explore gambling behavior, gambling problem severity, substance use, problem behavior, and mental health issues among minorities. Univariate analyses were conducted, comparing Whites (n = 1341) to respondents who identified as Hispanic (n = 394), Black (n = 261), or Asian/other (n = 177). Overall, the highest proportion of Hispanics were high-risk problem gamblers. Hispanic participants were also significantly more likely than other groups to use and abuse substances and to report mental health problems in the past month, behavioral addictions, and/or suicidal ideation in the past year. Primary predictors of White high risk problem gamblers were being young and male with friends or family who gambled, fair to poor health status, substance use, gambling once a week or more both online and in land-based venues, and engaging in a number of gambling activities. In contrast, gender was not a predictor of minority high risk problem gamblers, who were characterized primarily by having friends or family who gambled, gambling online only, having a behavioral addiction and playing instant scratch-offs and gaming machines. Implications for research and practice are discussed.

## Background

Studies have consistently reported high rates of problem gambling among racial and ethnic minorities compared to Whites, though findings differ by geographic location and socioeconomic status: ([Native American] Volberg and Abbott 1997; Zitzow 1996a, b; [Asian] (Marshall et al. 2009; Petry et al. 2003; Toyama et al. 2014); [Hispanic or Latino] Barry et al. 2011a; Welte et al. 2001; [Black or African American] Barnes et al. 2009; Barry et al. 2011b; Welte et al. 2008).

A majority of studies focused on ethnicity investigated rates of gambling and problem gambling among Blacks, including African Americans. Results of a large nationally-representative study found that Blacks had twice the rate (2.2%) of disordered gambling compared to Whites and lower scores on general health measures; they were also more likely to be women in the lowest income brackets (Alegria et al. 2009). Similar findings have been reported regarding Black youth, who were significantly more likely than white youth to engage in heavy gambling (Barnes, et al. 2009). Overall, being young, male, and non-Hispanic Black was associated with high rates of gambling disorder in the U.S. National Comorbidity Survey Replication (NCS-R) data (Kessler et al. 2008). These findings generally mirror sociodemographic characteristics and comorbidity patterns found in earlier studies (Petry et al. 2005; Welte et al. 2001) as well as in special sub-groups of Black gamblers ([hotline callers] Barry et al. 2008; [casino self-excluders] Nower and Blaszczynski 2006; [homeless individuals] Nower et al. 2015; [veterans] Stefanovics et al. 2017). Welte et al. (2017) have noted that adults living in disadvantaged neighborhoods reported the most problem gambling symptoms, however studies have yet to explore the predictors of problem gambling versus other adaptive and maladaptive behaviors in these groups apart from religiosity, which serves as a protective factor (Welte et al. 2017).

There is scant research involving Hispanics/Latinos and gambling. The few studies that exist are small-scale investigations of specific sub-groups. One general population survey reported that Hispanics/Latinos with subthreshold gambling problems were more likely to have comorbid mood, anxiety, substance use, and personality disorders than White participants. In another study of Latino American veterans, Westermeyer et al. (2005) found that the lifetime prevalence rate of disordered gambling was 4.3%, nearly four times higher than in the general population. The study further noted that gambling disorder was comorbid with high rates of major depressive (14.1%), alcohol (22.9%), and posttraumatic stress (12.2%) disorders in that sample. More than half of the undocumented Mexican immigrants surveyed in a small study in New York City reported having gambled, and a majority of those gamblers played scratch and win tickets or the lottery (Momper et al. 2009). Those who sent money home to their families or had lived in the United States more than 12 years and those who reported 1–5 days of poor mental health in the past 30 days were most likely to gamble.

Research among Asian gamblers has been limited, possibly because of the tension between the permissive attitude toward gambling and the increased stigma ascribed to those who gamble problematically in Asian groups (Dhillon et al. 2011). In the U.S., studies have identified higher rates of gambling and problem gambling among Asian subgroups, such as Southeast Asian and Cambodian refugees in the U.S., who reported rates of gambling disorder as high as 59% (Petry et al. 2003) and 13.9% (Marshall et al. 2009), respectively. Similarly, another study found that, among college students, Chinese students reported the highest rates of gambling problems followed by Koreans then Whites. The most significant predictors of problem gambling in that study were being Chinese or Korean and male, and having an alcohol or drug problems (Luczak and Wall 2016).

The culturally-based motivation to gamble and the risk and protective factors that fuel or arrest the progression toward problem gambling in ethnic sub-groups are likely complex and varied. Some researchers have suggested that the stress of acculturation may play a significant role. A recent study, examining differences in gambling behavior among first, second, and third generation immigrants from a diverse collection of world regions (Africa, Asia, Europe, and Latin America), found the lowest rates of gambling participation among Latin Americans, followed by Africa, Asia, and Europe, which had the highest rates. First-generation immigrants had lower rates of gambling prevalence and problem gambling when compared to second and third generation immigrants or native-born Americans. In addition, the study found that immigrants who arrived in the U.S. as children (12 or younger) gambled more frequently than those arriving as adolescents or adults (Wilson et al. 2015). Issues surrounding acculturative stress may also play a role in the development of gambling problems among youth. A recent study found that rates of at-risk or problem gambling among first generation adolescent immigrants were twice as high as their non-immigrant peers, particularly if they lived apart from their parents (Canale et al. 2017).

In addition to the influence of acculturation, other theorists have suggested that biology, values and beliefs also play a role. Chamberlain et al. (2016) suggested that inflated rates of problem gambling among some ethnic and racial groups may be due, in part, to neurocognitive differences among groups, as measured by differing rates of compulsivity, errors on memory and set-shifting tasks, and delay aversion, which they found were higher in Black versus White participants in one study. Other researchers underscore the influence of values and beliefs inherent in specific cultural groups or sub-groups in the progression and maintenance of problem gambling behavior (Alegria et al. 2009; Raylu and Oei 2004; Sacco et al. 2011). For example, certain Asian cultures consider gambling activities to be a part of their lifestyle and tradition (Clark et al. 1990; Raylu and Oei 2004). In other ethnic groups and cultures (e.g. Native Americans), the concepts of fate and a reliance on magical thinking may encourage gambling behavior in the same way as cognitive distortions do in pathological gamblers (Hardoon et al. 2001; Zitzow 1996a, b). Issues of social isolation, language barriers, and access to employment must also be clinically considered as factors which can drive immigrant populations towards pathological gambling behavior (Ngai and Chu 2001; Tse 2003).

To date, a notable exception has been found in the Hispanic native born and immigrant communities where, despite the adversity of poverty, lack of education, and social discrimination, rates of pathological and problem gambling are below that of the White majority (Alegria et al. 2009). This phenomenon seems to parallel the “Hispanic paradox” (Scribner 1996) documented in health outcome studies, where Hispanics have better health outcomes despite the challenges of low socioeconomic status and barriers to accessing healthcare (Grant et al. 2004; Scribner 1996; Vega et al. 1998).

Given the lack of clarity surrounding differences among minority groups and between minority and White gamblers, the purpose of this study is to explore differences in the characteristics and behaviors of non-problem gamblers compared to high-risk problem gamblers across different ethnic groups.

## Methods

### Participants

The study utilized a sub-set of 2173 New Jersey residents over 18 who endorsed at least one gambling activity in the past year from a larger epidemiologic study of 3634 participants. The remaining 1461 participants reported no involvement in any gambling activities in the past year and were excluded from the analyses. Data coding and analyses were conducted using SPSS version 24.

### Measures

The present study incorporated data collected through an epidemiological survey conducted across the state of New Jersey that stratified its sampling method to accurately reflect the demographic makeups of each region of the state. Sections of the survey produced data on the following variables: (a) demographics (gender, age, race/ethnicity, education level, household income, immigration status, and relationship status); (b) substance use (tobacco use, alcohol use, illegal drug use, problems and treatment seeking with substances, behavioral addictions, and binge drinking); (c) mental health and physical health (overall stress level, overall level of happiness, overall health, experiences of mental health problems in the past 30 days and 12 months, suicidal ideation, and suicidal attempts in the past year); (d) gambling activities participated in the past year (lottery, bingo, scratch offs, sports betting, horse race track betting, poker, casino table games, other games of skill, and gaming machines); (e) non-gambling activities participated in the past year (high risk stocks and daily fantasy sports); (f) gambling behavior (frequency of participation, amount of money spent, venue preference for gambling, and online gambling participation across all previously mentioned forms).

*Problem Gambling Severity Index (PGSI) of the Canadian Problem Gambling Index (CPGI, Ferris and Wynne* 2001 *)* This 9-item instrument was used to assess gambling status. Respondents indicate the extent to which an item applies to them using a four-point Likert scale ranging from 0 (never) to 3 (almost always). Scores are totaled in accordance with Ferris and Wynne’s (2001) guidelines: 0 indicates no risk; 1–2 low risk; 3–7 moderate risks; and 8–27 problem gambling, respectively. Ferris and Wynne (2001) reported satisfactory scale reliability (α = 0.84). For the purpose of the logistic regression analyses, a non-problem gambler was classified as any scoring 0 on the PGSI and “at-risk” gamblers were classified as any participant scoring 3 or higher on the PGSI.

### Procedure

The data was collected both by telephone (cell and landline phones) and Internet to address limitations inherent in either methodology alone. Stratified sampling was used in both sub-samples to ensure demographic characteristics of age, gender, and race/ethnicity were reflective of the New Jersey population.

## Results

### Univariate analyses

Univariate comparisons among problem severity categories were performed for gender, age, race/ethnicity, education level, marital status, household income, and employment status. Table 1 presents the distribution and statistical significance of explanatory variables by PGSI category. The association between the PGSI and each explanatory variable was assessed using Chi-squared Test of Independence. No socioeconomic variables showed a significant association with the PGSI. High risk of problem gambling was significantly associated with age (younger), gender (male), race/ethnicity (Hispanic and Asian/other), marital status (married), self-assessed health in the past year (Excellent), and past year stress (high). Non-problem gambling was significantly associated with age (older), gender (female), race/ethnicity (White), marital status (divorced/separated), self-assessed health in the past year (good/fair) and past year stress (low).

**Table 1:** Demographic breakdown of non-problem (n = 1510) and at-risk problem gamblers (n = 663)

Variable	Non-PG		Low risk PG		Moderate risk PG		High risk PG		Total
	*n*	%	*n*	%	*n*	%	*n*	%	*n* (% of total)
Age*									
21–24	92	6.1	34	12.3	28	14.8	38	19.4	192 (8.8)
25–34	237	15.7	65	23.5	52	27.5	73	37.2	427 (19.6)
35–44	312	20.6	49	17.7	50	26.5	55	28.1	466 (21.4)
45–54	332	22.0	66	23.8	32	16.9	21	10.7	451 (20.8)
55–64	243	16.1	30	10.8	12	6.3	7	3.6	292 (13.4)
65+	295	19.5	33	11.9	15	7.9	2	1.0	345 (15.9)
Gender*									
Male	695	46.0	150	54.2	120	63.2	136	69.4	1101 (50.6)
Female	815	54.0	127	45.8	70	36.8	60	30.6	1072 (49.4)
Race/ethnicity*									
White	1016	67.3	155	60.0	90	47.4	80	40.8	1341 (61.7)
Hispanic	245	16.2	40	14.4	49	25.8	60	30.6	394 (18.1)
Black	155	10.3	51	18.4	27	14.2	28	14.3	261 (12.0)
Asian/other	94	6.2	31	11.2	24	12.6	28	14.3	177 (8.2)
Marital status*									
Married or living w/partner	937	62.0	162	58.5	108	56.8	139	70.9	1346 (62.0)
Divorced, separated, Widowed	241	16.0	42	15.2	15	7.9	19	9.7	317 (14.6)
Single (never married)	332	22.0	73	26.3	67	35.3	38	19.4	510 (23.4)
Health status (past year)*									
Excellent	271	17.9	35	12.6	38	20.0	59	30.1	403 (18.5)
Good/fair	1051	69.6	197	71.2	118	62.1	111	56.6	1477 (68.0)
Poor	188	12.5	45	16.2	34	17.9	26	13.3	293 (13.5)
Overall stress level (past year)*									
Low	355	23.5	56	20.2	37	19.5	30	15.3	478 (22.0)
Moderate	1020	67.6	200	72.2	137	72.1	121	61.7	1478 (68.0)
High	135	8.9	21	7.6	16	8.4	45	23.0	217 (10.0)
*Yearly household income*									
Less than $15,000	65	4.3	14	5.1	9	4.7	13	6.6	101 (4.7)
$15,000–29,999	137	9.1	19	6.9	30	15.8	18	9.3	204 (9.4)
$30,000–49,999	207	13.7	53	19.1	28	14.7	21	10.7	309 (14.2)
$50,000–69,999	256	17.0	54	19.5	42	22.1	44	22.4	396 (18.2)
$70,000–99,999	305	20.2	57	20.6	36	18.9	41	20.9	439 (20.2)
$100,000–124,999	198	13.1	36	13.0	18	9.6	34	17.3	286 (13.2)
$125,000–149,999	120	7.9	15	5.4	10	5.3	14	7.2	159 (7.3)
$150,000 or more	222	14.7	29	10.4	17	8.9	11	5.6	279 (12.8)
*Education level*									
Less than high school or GED	17	1.1	10	3.6	5	2.6	12	6.1	44 (2.0)
High school diploma or GED	294	19.5	60	21.7	34	18.0	33	16.8	421 (19.4)
Some college (less than 1 year)	114	7.5	30	10.8	23	12.1	18	9.2	185 (8.5)
Some college (more than 1 year)	187	12.4	35	12.6	19	10.0	15	7.7	256 (11.8)
Associate’s degree	145	9.6	17	6.1	15	7.9	22	11.2	199 (9.1)
Bachelor’s degree	465	30.8	89	32.1	55	28.9	44	22.4	653 (30.1)
Master’s degree	219	14.5	27	9.7	25	13.2	33	16.8	304 (14.0)
Professional degree	38	2.5	6	2.2	9	4.7	13	6.6	66 (3.0)
Doctorate degree	31	2.1	3	1.2	5	2.6	6	3.2	45 (2.1)
*Employment status*									
Employed for Wages	843	55.8	173	62.5	120	63.2	127	64.7	1263 (58.2)
Self-employed	121	8.0	24	8.7	15	7.9	25	12.7	185 (8.5)
Out of work (less than 1 year)	34	2.3	5	1.8	2	1.1	7	3.6	48 (2.2)
Out of work (more than 1 year)	32	2.1	7	2.5	9	4.7	5	2.6	53 (2.4)
Homemaker	90	6.1	14	5.1	6	3.2	12	6.1	122 (5.6)
Student	61	4.0	15	5.3	17	8.9	7	3.6	100 (4.6)
Retired	283	18.7	31	11.2	15	7.8	6	3.1	335 (15.4)
Unable to work	46	3.0	8	2.9	6	3.2	7	3.6	67 (3.1)

* p ≤ .01

Additionally, Table 2 presents associations between race/ethnicity and gambling frequency, preferred gambling venue(s), participation in individual gambling activities, five measures of substance use, and three measures of mental health. Race/ethnicity was significantly associated with both high (Hispanics) and low frequency (Whites) gambling, land-based only gambling (Whites), and gambling both online and in land-based venues (Hispanics). Looking at specific gambling activities, race/ethnicity was significantly associated with instant scratch-off ticket play, bingo, sports betting, horse race track betting, live poker, live casino table games and other games of skill. Asians were more likely than other ethnicities to have participated in bingo within the past year, while Hispanics preferred sports betting, horse race track betting, live poker games, live casino table games and other games of skill. Hispanic participants were distinguished by their answers to questions pertaining to substance use and mental health issues. Hispanic respondents were more likely than the other ethnicities to endorse tobacco use, binge drinking, illegal drug use and problems due to drug or alcohol use in the past year. Hispanic participants were also more likely than other groups to endorse a mental health problem in the past 30 days, having a behavioral addiction and/or suicidal ideation in the past year.

**Table 2:** Gambling, substance use, and mental health by ethnicity

Variable	White		Hispanic		Black or African American		Asian/other		Total
	*n* (1341)	%	*n* (394)	%	*n* (261)	%	*n* (177)	%	*n* (% of total)
Gambling frequency**									
Low	478	66.1	114	15.8	75	10.4	56	7.7	723 (100.0)
Medium	367	63.1	92	12.7	81	13.9	42	7.2	582 (100.0)
High	496	57.1	188	21.7	105	12.1	79	9.1	868 (100.0)
Preferred gambling venue(s)***									
Land-based only	1067	66.2	244	15.1	198	12.3	104	6.4	1613 (100.0)
Online only	66	57.4	26	22.6	7	6.1	16	13.9	115 (100.0)
Land-based and online	208	46.7	124	27.9	56	12.6	57	12.8	445 (100.0)
*Gambling activities*									
Lottery	1059	60.7	323	18.5	219	12.6	143	8.2	1744 (100.0)
Instant scratch-off tickets**	853	60.6	276	19.6	181	12.9	98	7.0	1408 (100.0)
Bingo***	212	51.0	92	22.1	55	13.2	57	13.7	416 (100.0)
Sports betting***	139	43.3	98	30.5	42	13.1	42	13.1	321 (100.0)
Horse race track betting***	201	61.9	75	23.1	18	5.5	31	9.5	325 (100.0)
Live poker***	129	51.2	70	27.8	26	10.3	27	10.7	252 (100.0)
Live casino table games***	264	57.0	104	22.5	42	9.1	53	11.4	463 (100.0)
Gaming machines (slots)	416	60.4	139	20.2	73	10.6	61	8.8	689 (100.0)
Other games of skill***	158	45.7	99	28.6	47	13.6	42	12.1	346 (100.0)
*Substance use*									
Tobacco use***	351	53.6	156	23.8	93	14.2	55	8.4	655 (100.0)
Alcohol use***	1051	62.2	325	19.2	178	10.5	136	8.0	1690 (100.0)
Binge drinking***	230	51.7	120	27.0	45	10.1	50	11.2	445 (100.0)
Illegal drug use***	116	44.3	82	31.3	41	15.6	23	8.8	262 (100.0)
Problems with drugs or alcohol***	44	40.4	43	39.4	13	11.9	9	8.9	109 (100.0)
*Mental health*									
Behavioral addictions*	165	55.9	74	25.1	35	11.9	21	7.1	295 (100.0)
Mental health problems*	183	60.4	70	23.1	35	11.6	15	5.0	303 (100.0)
Suicidal ideation***	33	44.0	26	34.7	11	14.7	5	6.7	75 (100.0)

* p ≤ .05; ** p ≤ .01; *** p ≤ .001

### Multivariate analyses

A primary aim of this study was to identify the primarily predictors of those at moderate or high risk for gambling problems (i.e. 3+ symptoms) compared to non-problem gamblers (i.e. zero symptoms). For that reason, medium and high risk participants were recoded as “problem gamblers” and compared to non-problem gamblers. Low risk gamblers were omitted from the analyses to ensure comparisons between those with more serious symptoms to those with an absence of symptoms. Multiple logistic regression analyses were used to evaluate the relative contributions of the predictor variables, which had proven significant in the univariate analyses, to the likelihood of membership in the at-risk problem gambling group. Continuous variables included age and number of gambling activities endorsed for the past year. All other variables were dummy coded. The minimum criteria for entry of covariates into the model were a *p* value of less than .05. Partial odds ratios (OR) and 95% confidence intervals (CIs) were computed for significant predictors. Model effects were estimated by the improvement in Chi-square and by a classification matrix indicating the proportion of individuals correctly identified by the model covariates.

To facilitate the identification of specific demographic, mental health, gambling participation, and substance use characteristics that differentiate non-problem gamblers from problem gamblers in Whites and ethnic minorities, backward selection step-wise logistic regression analyses were performed, entering in Block 1 demographic variables that had proven significant in the prior analyses between the two groups. These included gender, age, marital status, whether friends or family gamble, overall health in the past year, and overall stress levels in the past year. Substance use, behavioral addiction, and mental health variables were entered in Block 2, to determine which of the significant variables added most to the regression equation overall and which, if any, had a moderating effect on the significant demographic characteristics. Gambling behavior variables were entered into Block 3 to similarly determine which added the most to the regression equation overall and had a moderating effect on the remaining Block 1 and Block 2 variables. Tables 3 and 4 show the final regression results.

**Table 3:** Variables distinguishing between White non-problem gamblers (n = 1016) and White at-risk gamblers (n = 325)

	SE	*OR*	95% CI
Age (continuous)***	0.01	0.98	0.97–0.99
Gender (female)*	0.17	1.44	1.03–2.02
Friends and family gamble***	0.17	2.28	1.64–3.18
*Health status for the last year* *Excellent (ref.)*			
Fair**	0.31	2.69	1.46–4.94
Poor*	0.25	1.64	1.00–2.69
Tobacco use**	0.18	1.73	1.22–2.44
Alcohol use	0.21	0.20	0.50–1.15
Binge drinking	0.21	1.50	0.99–2.26
Problems with drugs or alcohol*	0.51	2.77	1.03–7.47
Behavioral addictions**	0.23	1.84	1.16–2.91
*Gambling frequency* *Low (ref.)*			
Medium*	0.23	1.70	1.08–2.68
High***	0.22	2.80	1.83–4.29
*Gambling venue* *Land-based only (ref.)*			
Online and land-based***	0.23	2.74	1.76–4.26
Online only**	0.33	2.55	1.35–4.81
Instant scratch-off***	0.20	2.72	1.83–4.04
Sports betting**	0.28	2.35	1.36–4.05
Horse race track	0.25	.66	0.40–1.08
Live casino table games*	0.21	1.65	1.10–2.47
Other games of skill*	0.24	1.75	1.09–2.81
Gaming machines*	0.18	1.59	1.12–2.27

* *p* ≤ .05; ** *p* ≤ .01; *** *p* ≤ .001

**Table 4:** Variables distinguishing between Minority non-problem gamblers (n = 494) and Minority at-risk problem gamblers (n = 338)

	SE	*OR*	95% CI
Age (continuous)*	0.01	0.98	0.97–1.00
Gender (female)	0.20	0.68	0.74–1.60
Friends and family gamble***	0.19	2.95	2.04–4.26
*Overall stress level in the past year* *Low (ref.)*			
Moderate	0.24	1.29	0.81–2.05
High	0.39	1.08	0.50–2.31
*Relationship status* *Married (ref.)*			
Divorced, separated, or widowed	0.31	1.02	0.56–1.88
Single	0.22	0.86	0.56–1.32
Tobacco use	0.21	1.42	0.96–2.16
Binge drinking	0.22	1.33	0.87–2.03
Illegal drug use	0.27	1.58	0.90–2.59
Behavioral addictions**	0.28	2.16	1.26–3.86
Suicidal ideation in the past year	0.61	1.61	0.46–5.20
*Gambling frequency low (ref.)*			
Medium***	0.28	3.60	2.08–6.24
High***	0.27	4.53	2.67–7.70
*Gambling venue* *Land-based only (ref.)*			
Online and land-based	0.24	1.53	0.96–2.44
Online only*	0.38	2.47	1.17–5.21
Instant scratch-off*	0.22	1.63	1.06–2.50
Bingo	0.25	1.54	0.95–2.49
Sports betting	0.28	1.63	0.95–2.81
Live casino table games	0.26	1.56	0.95–2.58
Gaming machines*	0.22	1.55	1.02–2.36

* *p* ≤ .05; ** *p* ≤ .01; *** *p* ≤ .001

The results of both logistic regressions indicated a good model fit. The regression model separating White non-problem gamblers and at-risk problem gamblers presented with a Hosmer–Lemeshow goodness-of-fit statistic of, χ^2^ (8, N = 1341) = 2.91, p = .940. The second regression model separating ethnic minority non-problem gamblers and at-risk problem gamblers presented with a Hosmer–Lemeshow goodness-of-fit statistic of, χ^2^ (8, N = 832) = 10.25, p = .248. The largest predictors for membership in the White at-risk problem gambler group in the final model were high frequency gambling, having problems with drugs or alcohol, gambling both online and in land-based venues, and participating in instant scratch-off tickets. The largest predictors for membership in the minority at-risk problem gamblers group in the final model were high and moderate frequency gambling, having friends or family that gamble, and gambling online only.

Among Whites, the results indicate a significant negative relationship with age: Each one-year increase in age decreased the odds of being an at-risk problem gambler by .98%. Men were 1.44 times more likely to be White at-risk problem gamblers in comparison to women. Having friends or family who gambled increased the odds of being a White at-risk problem gambler by 2.28 times. Whites were also characterized by fair (2.69 times) or poor (1.64 times) health status in the past year, using tobacco products (1.73 times), having problems with drugs or alcohol (2.77 times) and/or a behavioral addiction (1.84 times).

Among Whites, high frequency (2.8 times) or moderate frequency (1.7 times) gambling, gambling online (2.6 times) or both online and in land-based venues (2.7 times), purchasing scratch-off tickets (2.7 times), betting on sports (2.3 times), playing games of skill (1.8 times), live casino games (1.7 times) and/or gaming machines (1.6 times) were most predictive of at-risk problem gamblers.

Among ethnic minorities, there was a similar negative relationship with age: Each one-year increase decreased the odds of being an at-risk problem gambler. Gender was a non-significant predictor for minorities, although having friends or family that gambled proved the most significant predictor for minority at-risk problem gambling status, increasing the odds by nearly three times. Among the substance use and mental health variables, only having a behavioral addiction was significant predictor of at-risk problem minority membership, increasing the odds by 2.0 times. As with Whites, moderate or high frequency gambling increased the odds of being an at-risk problem gambler by 3.6 and 4.5 times, respectively. Unlike Whites, however, gambling both online and in land-based venues was not a significant predictor of being at-risk, although gambling only online increased the odds of membership by 2.5 times. Amongst the individual gambling activities, only instant scratch-off tickets and gaming machine participation were predictive of at-risk minority status (2.72 and 1.59 times respectively).

## Discussion

Findings from this study highlight the need to further explore ethnic differences among gamblers and to better differentiate etiological and other risk factors that may variously predispose different ethnic groups to develop gambling problems. The study utilized a representative sample of participants from New Jersey, however, the relatively small sample size of each ethnic sub-group compared to Whites precluded a detailed exploration of differences within each sub-group in the multivariate analyses. The data suggested that, overall, Whites were more likely than other ethnic groups to be non-problem gamblers; they were also more likely than other ethnic groups, irrespective of problem gambling severity, to be younger males from families or peer groups that gambled and to report comorbid addictive behaviors and fair to poor health status. This profile reflects the characterization of the “emotionally vulnerable” problem gambler (Blaszczynski and Nower 2002), who gambles problematically in order to escape aversive mood states and develops problems due to gambling with increasing frequency on multiple gambling games. Like Whites, Ethnic minority groups appear to be primarily influenced by family members or peer groups who gambled, however, unlike Whites, gender did not appear to play a predictive role. As with Whites, higher gambling frequency among minorities was correlated with higher levels of problem severity, although gambling only online and presumably on gaming machines appeared to be a greater risk factor. These findings could suggest that the influence of cultural, familial and community attitudes about gambling, combined with accessibility of opportunities and the conditioning effects of reinforcement could lead to gambling problems in some minority subgroups. This etiology, characteristic of “behaviorally conditioned” problem gamblers (Blaszczynski and Nower 2002), is most responsive to targeted prevention, interventions, and education efforts directed at the client system.

In contrast to findings in an earlier study (Alegria et al. 2009), the current results fail to support the notion of a “Hispanic paradox” for gambling and suggest a far more complex and context-dependent array of risk factors likely play a role. In this study, Hispanics were distinguished by the highest rates of problem gambling, substance abuse, and mental health problems. Though Asian participants also endorsed high rates of problem gambling, Hispanic gamblers reported the highest proportionate rates of “action” oriented play, such as sports and race track betting and casino table games, and gambling primarily online. They were also more likely than other ethnic groups to endorse substance abuse, mental health problems and suicidality in the past year.

Very little is known about the onset of gambling and problem gambling in Hispanic communities, the influence of peers and family modeling, the role of erroneous cognitions generated by cultural superstitions, and/or other bio-psycho-social factors that lead to the development and maintenance of gambling problems in sub-groups of Hispanics and Latinos. In New Jersey, Hispanics are the largest minority but their median income is almost half that of Whites and less than half that of Asians (U.S. Census Bureau 2015), however, there are few programs and services targeting Hispanic gamblers and few certified gambling counselors who are Spanish-speakers. Future research with Hispanics and other ethnic minorities should focus on exploring the cultural and familial systems that introduce and help to maintain gambling behavior in various ethnic groups and investigating specific risk and protective factors to use as a basis for prevention, intervention and treatment efforts.
